# A Novel Passive Wireless Sensor for Concrete Humidity Monitoring

**DOI:** 10.3390/s16091535

**Published:** 2016-09-20

**Authors:** Shuangxi Zhou, Fangming Deng, Lehua Yu, Bing Li, Xiang Wu, Baiqiang Yin

**Affiliations:** 1 School of Civil Engineering and Architecture, East China Jiaotong University, Nanchang 330013, China; zhoushuangxi@ecjtu.jx.cn (S.Z.); yulehua@ecjtu.jx.cn (L.Y.); 2 School of Electrical and Automation Engineering, East China Jiaotong University, Nanchang 330013, China; zgxiangyu@163.com; 3 School of Electrical Engineering and Automation, Hefei University of Technology, Hefei 230009, China; libinghnu@hfut.edu.cn (B.L.); yinbaiqiang123@hfut.edu.cn (B.Y.)

**Keywords:** wireless humidity sensor, RFID technology, concrete humidity measurement, rectifier, regulator, patch antenna

## Abstract

This paper presents a passive wireless humidity sensor for concrete monitoring. After discussing the transmission of electromagnetic wave in concrete, a novel architecture of wireless humidity sensor, based on Ultra-High Frequency (UHF) Radio Frequency Identification (RFID) technology, is proposed for low-power application. The humidity sensor utilizes the top metal layer to form the interdigitated electrodes, which were then filled with polyimide as the humidity sensing layer. The sensor interface converts the humidity capacitance into a digital signal in the frequency domain. A two-stage rectifier adopts a dynamic bias-voltage generator to boost the effective gate-source voltage of the switches in differential-drive architecture. The clock generator employs a novel structure to reduce the internal voltage swing. The measurement results show that our proposed wireless humidity can achieve a high linearity with a normalized sensitivity of 0.55% %RH at 20 °C. Despite the high losses of concrete, the proposed wireless humidity sensor achieves reliable communication performances in passive mode. The maximum operating distance is 0.52 m when the proposed wireless sensor is embedded into the concrete at the depth of 8 cm. The measured results are highly consistent with the results measured by traditional methods.

## 1. Introduction

Structural Health Monitoring (SHM) systems are automated tools, aimed at rapidly identifying the onset of structural damage and at tracking the condition of structures during forced or natural excitation [1]. Current SHM systems almost adopt Non-Destructive Testing (NDT) techniques, which can be divided into two types: on-demand type and in situ. The on-demand NDT techniques are based on instrumentation brought to the measurement site on-demand, including X-ray, infrared thermography, laser scanning and microwave radar [2,3,4,5]. However, these techniques cannot measure the physical quantities of concrete directly and the measured results are not real-time. The second family is based on the deployment of several local sensors permanently coupled with structural elements. The in situ techniques can also be divided into two groups, according to the use of wired or wireless technology. Among the wired ones [6,7], high measuring precision, great resolution, and real time monitoring can be obtained, however they are quite time-consuming and expensive because of the complicated manufacturing as well as the extensive signal processing.

Thanks to fast and easy installation, wireless sensors are gaining a growing interest. Most of the wireless sensors for SHM are based on Wireless Sensor Networks (WSN), which are battery-powered and permit long-range operating [8,9]. However, once these wireless sensors are immersed in concrete, their batteries cannot be replaced again and their lifespan are limited to several years. Furthermore, their cost and complexity are still relatively high. Recently, with the rapid development of Radio Frequency Identification (RFID) technology, the wireless sensors based on passive RFID technology have aroused great interest [10]. The passive tag is able to passively communicate with the interrogator on a zero-powered backscatter mechanism, resulting in simple architecture, low power, and low cost. The passive RFID sensors are excellent choices for long-term and low-cost SHM [11,12].

Humidity is one of the parameters considered as a critical factor in the progress of cement maturity by influencing the stability, the transformation of hydrates, and strength development [13]. The result of this process is an increased strength and decreased permeability. Uncontrolled moisture diffusion during the setting and curing process can cause a number of detrimental effects. For example, fast moisture diffusion resulting from high temperature during curing, may lead to higher shrinkage stresses and lower long-term concrete strength [14,15]. An insufficient water supply due to this diffusion and the evaporation of the internal moisture will hinder the hydration of cement [16]. Besides, during the process of casting reinforced concrete, the corrosion of steel reinforcing bars due to high humidity will decrease the service life of reinforced concrete structures [17]. Therefore, humidity monitoring is significant for concrete.

There are various kinds of RFID-based humidity sensors. The simplest kind is the chipless RFID temperature sensor, which requires no Integrated Circuits (ICs) and transmits sensor data by changing the radar cross section of the RFID antenna [18]. The characters of transmitting analog sensor data and no digital blocks lower its performances and constrain its applications mainly in low-cost fields. In situations where there are high accuracy requirements or a large number of sensors in close proximity, chip-based RFID sensors are employed to incorporate digital modulation. A humidity-to-frequency sensor in CMOS technology with wireless readout is presented in [19], however this wireless humidity sensor only achieves a working range of 30 mm because of 13.56 MHz operating frequency. A wireless temperature and humidity sensors based on Ultra-High Frequency (UHF) is proposed in [20]. Because of the complex architecture and off-chip humidity sensor, the wireless humidity sensing range in air is only 2.7 m, which cannot meet the tough requirements of concrete monitoring.

This paper presents a novel wireless humidity sensor for SHM. To our knowledge, this is the first work to measure the concrete humidity based on UHF RFID technology. The rest of the paper is organized as follows. Section 2 analyzes electromagnetic wave transmission in concrete and proposes a novel architecture of wireless humidity sensor for ultra-low power application. Section 3 presents the detailed design of key blocks. Section 4 illustrates the measurement results and compared it with the results of traditional measurement. The conclusion is made in Section 5.

## 2. System Design of Proposed Wireless Humidity Sensor

**Figure 1:**
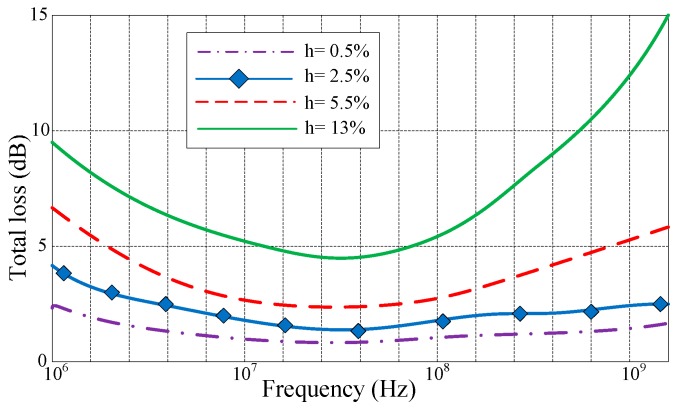
Total loss of electromagnetic wave penetrating concrete.

Since concrete has distinct electromagnetic properties at different humidity conditions, the total power loss for electromagnetic waves penetrating concrete are analyzed through the 1 MHz to 1 GHz frequency range for various humidity conditions (at depth of 0.2 m), i.e., 0.5%, 2.5%, 5.5%, and 13%. The matlab simulation results are shown in Figure 1, which are calculated from the ratio of water to the volume of the specimen [21,22,23]. As expected, due to the reverse variations of the transmission and propagation losses, an optimum frequency range exists, within which there is significantly smaller power loss. For example, the total loss in 20–80 MHz frequency range for wet concrete (13% humidity) is about 5 to 10 dB less than the total loss at either 1 MHz or 1 GHz. The proposed wireless sensor works at 915 MHz, the total loss is about 10 dB at 915 MHz, which is acceptable for concrete monitoring.

**Figure 2:**
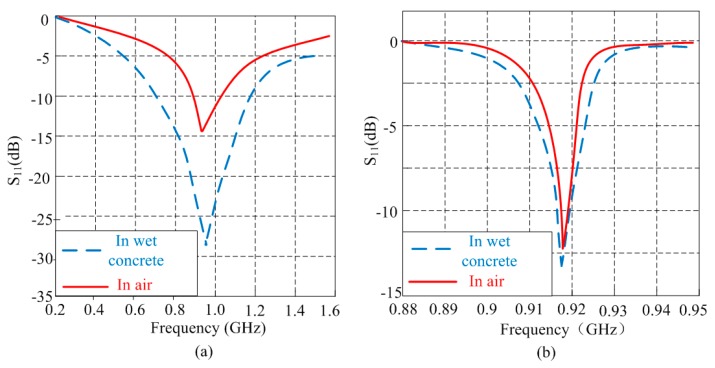
Computed S-parameters of two antennas coupling in free space and wet concrete: (**a**) return loss of dipoles; (**b**) return loss of patches.

The aim of the proposed RFID sensor is to perform humidity measurement inside the concrete, thus the type of the antenna should be taken into consideration. Figure 2 compares the return loss performances of the same RFID tag respectively equipped with two typical types of antenna (dipole and patch) in wet concrete and air. The results is based on the extended Debye model of concrete [24] and simulated by HFSS. The two pairs of RFID tags, one residing in air and another one residing inside the concrete slab at 8 cm depth, are designed to resonate at 915 MHz. From Figure 2a we can see the performance of dipole is dramatically affected when it is embedded in the concrete, the bandwidth is wider and the transmission loss increases obviously. However seen from Figure 2b, the performance of patch antenna is less sensitive to the humidity change of concrete. Hence, the patch antenna is more suitable for concrete monitoring.

The theoretical practicable operating power of an RFID tag *P_t_* is calculated from the Friis transmission equation [25]:

(1) $$P_{t}=E_{r}\cdot G_{a}\cdot \eta_{r}\cdot {\left(\frac{\lambda }{4\pi d}\right)}^{2}$$

where *E_r_* is the effective isotropic radiation power of a reader, *G_a_* is the tag antenna gain, *η_r_* is the RF-to-DC power conversion efficiency of the rectifier, *λ* is the wavelength of the electromagnetic wave, and *d* is the communication distance. From Equation (1), the communication distance *d* can be expressed as:

(2) $$d=\frac{\lambda }{4\pi }\sqrt{\frac{E_{r}\cdot G_{a}\cdot \eta_{r}}{P_{t}}}$$

Hence, in order to achieve longer communication distance, lower *P_t_* and higher *η_r_* are critical for the design of UHF RFID tag, for *E_r_* is limited by regional regulations (4 W is the maximum transmitted power) and *G_a_* is roughly determined by the allowable antenna area (1.64 for the λ/2 dipole antenna).

**Figure 3:**
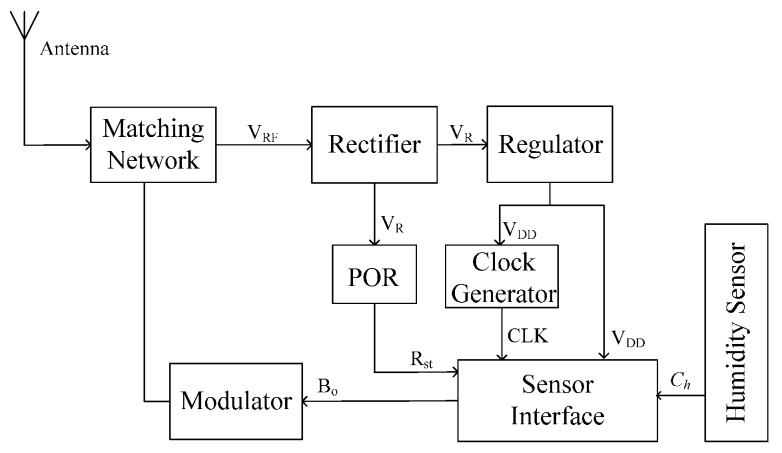
Architecture of the proposed wireless humidity sensor.

Because concrete is a high-loss material for electromagnetic wave transmission, this paper proposes a novel architecture to lower the overall power dissipation of wireless sensor for longer operating distance. Figure 3 shows the architecture of the proposed wireless humidity sensor based on UHF RFID technology. The blocks, except for the antenna and matching network, are integrated on a single chip. The sensor antenna, which is matched with the sensor chip through the matching network, receives the electromagnetic waves from the RFID reader. The rectifier multiplies and transfers the received RF signal to a DC supply voltage *V_R_* for the subsequent circuitry. Once the output of the rectifier reaches the operating voltage, the Power-On-Reset (POR) block generates a reset signal *R_st_* for the temperature sensor. The clock generator provides a reference clock CLK for the sensor interface. Because the sensor interface and the clock generator should operate under a stable supply voltage *V_DD_*, a regulator block is employed to stabilize the output voltage of the rectifier. This architecture does not include the demodulator and the baseband blocks in the traditional RFID tag, which means that this wireless humidity sensor will operate without any addressing as long as the sensor receives enough energy from the RFID reader [26].

## 3. Key Blocks Design

**Figure 4:**
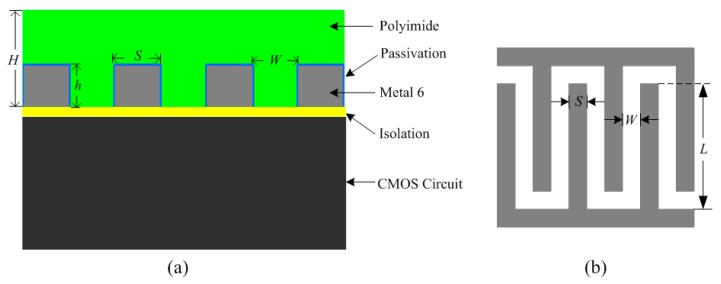
Proposed humidity sensor structure (**a**) humidity sensor structure; (**b**) top view of the humidity sensor

Inspired by the reported CMOS humidity sensor designs [27,28,29,30], interdigitated top metal fingers with polyimide filled into the finger gaps, can be utilized for capacitive humidity sensing. Figure 4a illustrates the structure of the proposed capacitive humidity sensor. The proposed humidity sensor was fabricated in the TSMC 0.18 μm 1P6M CMOS process. The top metal layer (Metal 6) was deposited and patterned with standard optical lithography and wet etching over the isolation layer to form the interdigitated structure. The sensing capacitor was then covered with a humidity-sensitive polyimide layer. The sensor is fabricated in a standard CMOS process without any post-processing. As seen from Figure 4b, *L* is the length of metal electrodes, *S* is the width of each electrode, and *W* is the distance between adjacent electrodes. The thickness of the polyimide layer is *H*, which is generally larger than metal thickness *h*. For a *N* finger array sensor, the total sensor capacitance *C_h_* can be expressed as [31]:

(3) $$C_{h}=N\varepsilon_{wet}\frac{Lh}{W}$$

where *L* is the length of metal electrodes, *h* is the thickness of metal layer, *W* is the distance between adjacent electrodes, and *ε_wet_* represents the dielectric constant of the polyimide film with absorbed water. Considering the factors including sensor capacitance, chip area sensitivity, etc., this work chooses *N* = 40, *L* = 200 µm, *W* = 2.5 µm, *S* = 2.5 µm, *h* = 1 µm, and *H* = 2 µm.

**Figure 5:**
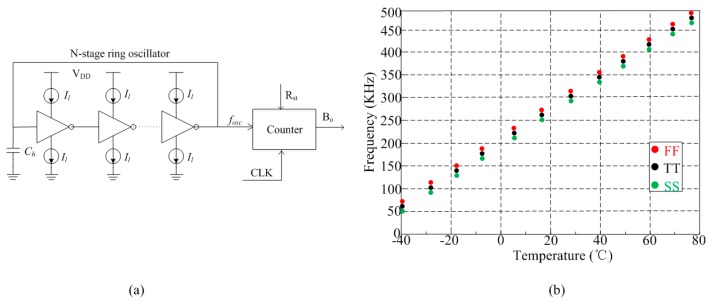
Proposed sensor interface: (**a**) architecture of sensor interface; (**b**) simulation results of *f_osc_* vs. temperature on different process corners.

The capacitive humidity sensor acts as a capacitor when it works with the sensor interface. The traditional interface starts with a capacitance-to-voltage converter, which is then followed by a voltage-to-digital converter [32,33]. This technique can achieve high speed and high resolution performances. However, due to the use of an operational amplifier, this technique consumes too much power. Figure 5a shows the architecture of proposed sensor interface, which converts the sensor signal in frequency domain for low-power application. The N-stage ring oscillator is employed to generate a controlled frequency *f_osc_* which is then countered by a 10-bit counter to output the corresponding digital output *B_o_*. For a standard ring oscillator, assuming an equal rise and fall time for the different stages respectively, the oscillating frequency of the ring oscillator *f_osc_* can be expressed as follows:

(4) $$f_{osc}=\frac{1}{t_{d}}=\frac{I_{l}}{V_{m}C_{l}}$$

where *t_d_* is the delay time of the loop, *I_l_* is the current flowing through the inverter, *C_l_* is the equivalent load capacitance of the loop, and *V_m_* is the swing range of the output voltage which mostly equals the supply voltage *V_DD_*. Because *C_l_* is mainly determined by the capacitance *C_h_* of humidity sensor, *f_osc_* is a sensor-controlled frequency and *B_o_* is the corresponding digital output of humidity sensor.

The simulation results of the proposed frequency *f_osc_* versus temperature on different process corners are shown in Figure 5b. The TT, FF, and SS corners correspond to “nominal”, “fast” and “slow” MOSFET devices, respectively. The *f_osc_* achieves good linearity with temperature and the simulated worst case variation across corners is around ±9%.

**Figure 6:**
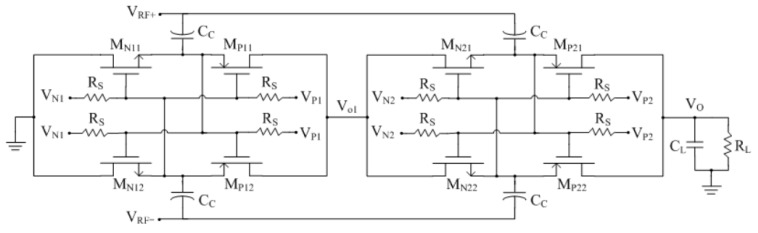
Schematic of the proposed two-stage rectifier.

A popular performance metric of a rectifier is its power conversion efficiency which is defined as:

(5) $$\eta_{r}=\frac{P_{out}}{P_{in}}$$

where *P_out_* is the average DC output power generated at the output of the rectifier and *P_in_* is the average RF power available at the input of the rectifier. From a circuit-level point of view, *η_r_* is mainly degraded due to the forward drop of the switch diodes or transistors [34]. Differed from the reported rectifier structure [35,36,37], Figure 6 shows the schematic of the proposed rectifier, which consists of two identical stages. The NMOS transistors M_N11–22_ and PMOS transistors M_P11–22_ form the differential-drive switch. In order to achieve a flat *η_r_* curve, the bias-voltage V_N1,2_ and V_P1,2_ are employed to boost the gate-source voltage of NMOS switch and PMOS switch, respectively [31]. The large resistor *R_S_* is added to block the AC component of V_N1,2_ and V_P1,2_. In CMOS process, *R_S_* could be replaced by a PMOS transistor that operates in the cut-off region to avoid the large silicon area.

**Figure 7:**
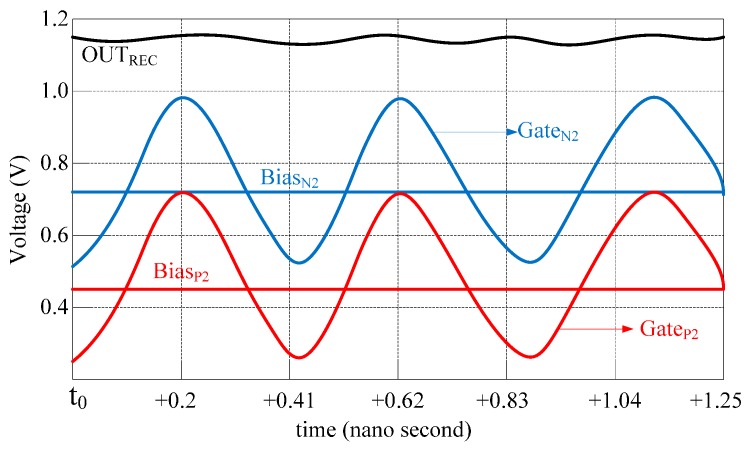
Second stage bias and gate voltages of the proposed gate boosting scheme.

Figure 7 depicts in more details how the proposed scheme sets the DC level of PMOS and NMOS gate voltages. The RF intermediate voltage (M_U_,_L_) is superimposed on the bias voltages Bias_N2_ and Bias_P2_ to provide the shifted voltages Gate_N2_ and Gate_P2_ to the gate of NMOS and PMOS switches, respectively.

**Figure 8:**
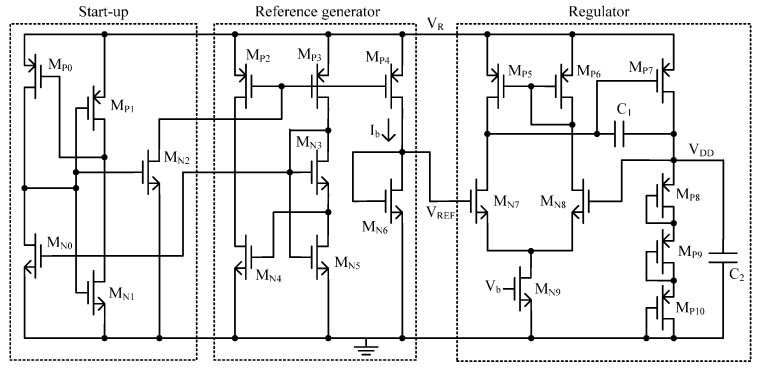
Proposed voltage regulator.

**Figure 9:**
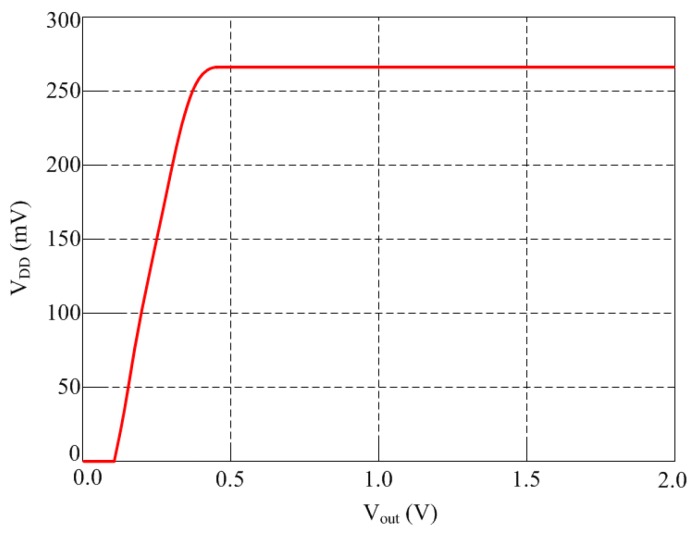
Simulated supply voltage variation of the proposed rectifier.

Due to the large variation of the output voltage of rectifier, a reference circuit with small supply voltage coefficient is necessary to generate a stable reference voltage. The traditional way to generate stable 1.25 V reference voltage is using a bandgap reference circuit [38]. However, it cannot meet the low-voltage requirement of low-power application. Figure 8 shows the schematic of the proposed voltage regulator. It is implemented in standard CMOS process without a bandgap reference and consists of three parts. The transisitors M_P0_–M_P1_ and M_N0_–M_N2_ compose a start-up block, which is added as a precautionary measure to ensure bias in the desired state. The transistors M_P2_–M_P4_ and M_N3_–M_N6_ compose a reference generator block to provide a reference voltage *V_REF_* of 0.26 V which is compensated by temperature and supply voltage. The transistors M_P5_–M_P10_ and M_N7_–M_N9_ compose a regulator to generate a stable voltage *V_DD_* of 1.0 V for other circuits. Figure 9 shows the *V_DD_* − *V_out_* characteristic at room temperature of 25 °C. The circuit starts working properly with *V_DD_* = 0.47 V. In the supply voltage range from 0.47 V to 2 V, an average reference voltage of 266.5 mV is generated. In this measured *V_DD_* range, the output voltage changes at most by 1.8 mV, thus leading to a line sensitivity of 0.441%/V.

**Figure 10:**
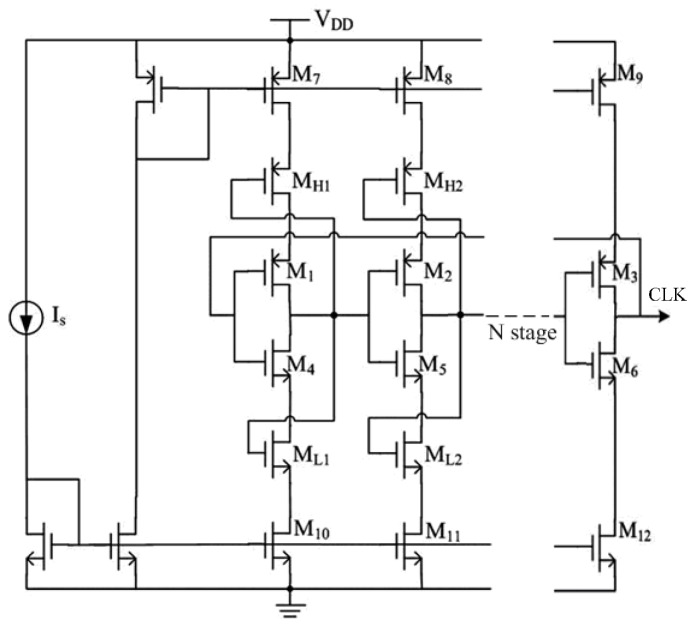
Schematic of the proposed clock generator.

The clock generator always employs ring oscillator architecture based on current-starved inverters. The output voltage of traditional current-starved inverters is close to the supply voltage and ground, which improves the output range while increasing the power consumption. As is shown in Figure 10, in order to achieve the balance between output range and power consumption, this paper proposed a novel structure of ring oscillator for the clock generator’s design. The PTAT oscillator discussed above in Figure 3 also adopts the same architecture. The transistors M_1_–M_6_ consist of an *N* stage current-starved inverter, the transistor M_7_–M_9_ and M_10_–M_12_ consist the current mirrors of inverter. Compared with the conventional structure, the extra transistors M_H1_, M_H2_, M_L1_, and M_L2_ are employed to reduce the voltage swing [39].

**Figure 11:**
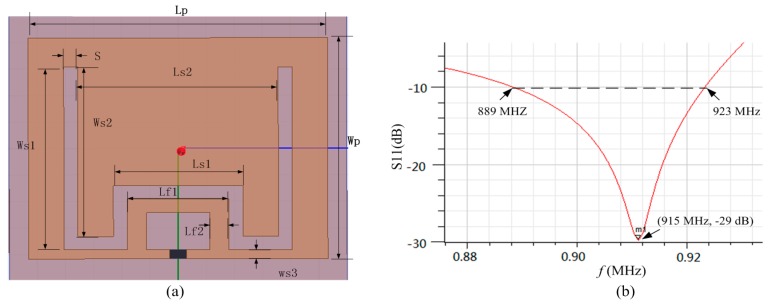
Proposed patch antenna: (**a**) antenna design; (**b**) return-loss plot.

Microstrip antenna is designed by including a ground plane structure, which has a natural anti-metal advantage compared to the dipole antenna. The length of rectangular microstrip patch antenna length is approximately equal to half of its electrical length, which is not suitable for the miniaturization of tags. As shown in Figure 11a, we presented a miniaturized microstrip antenna using the embedded short stub and U-type slot [40]. The impedance of chip was 19-j172 Ω at 915 MHz. The copper traces with the thickness of 0.035 mm were printed on a FR4 substrate, whose permittivity is 4.4 and thickness is 2 mm. The current in the short circuited stub of this antenna is the strongest, so the impedance of the antenna can be adjusted easily by adjusting the value of Lf1 and Lf2. The short stub embedded in radiation patch internal can lead to the reduction of the antenna volume. Furthermore, because the current can flow around the slot, it results in the increasing of the length of current path and further achieving the miniaturization purpose. The return-loss plot of this antenna is shown in Figure 11b. The return-loss at 915 MHz frequency is −29 dB and the bandwidth below −10 dB is from 889 MHz to 923 MHz. The gain of the proposed patch antenna is −3.2 dB and the maximum radiation direction is right above the antenna. The detailed parameters of the proposed patch antenna is shown in Table 1.

**Figure 12:**
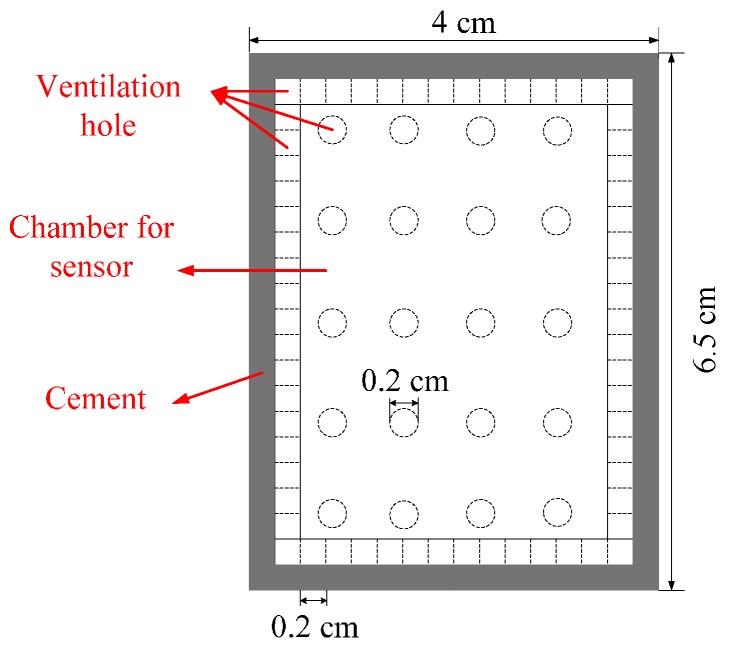
Top view of the proposed package box.

A protection device is necessary before the sensor tag is embedded into concrete, so that the sensor tag is not impaired by hydration heat arising from grouted concrete and not soaked directly in water of concrete. The transmission signal of sensor tag is sent out by electromagnetic wave, hence it is important to pay attention to wireless transmission impedance matching. Based on civil engineering techniques, this study proposes a protective high permeability acrylic package box. In order to keep the sensor in contact with the environment fully, 16 holes were punched on the side and 20 holes were punched at the bottom of the proposed package box. Figure 12 shows the top view of protection device. The package box covers an area of 4 × 6.5 mm^2^ and the diameter of the hole is 0.2 cm.

## 4. Experimental Characterization

**Figure 13:**
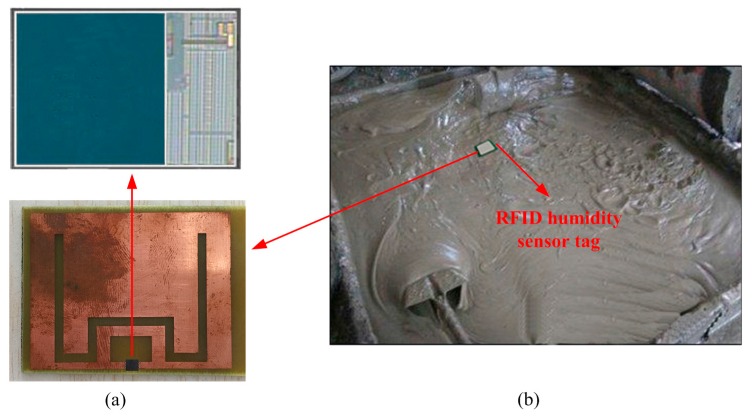
(**a**) Photo of the proposed tag chip; (**b**) Concrete measuring environment.

Figure 13a shows the proposed wireless humidity sensor, which was fabricated in the TSMC 0.18 μm CMOS process. The sensor tag chip covers around 8 × 3.5 mm^2^ and was equipped with this antenna on FR4 substrate by using flip chip. Then the sensor tag was packaged with the package box for concrete monitoring as shown in Figure 13b.

**Figure 14:**
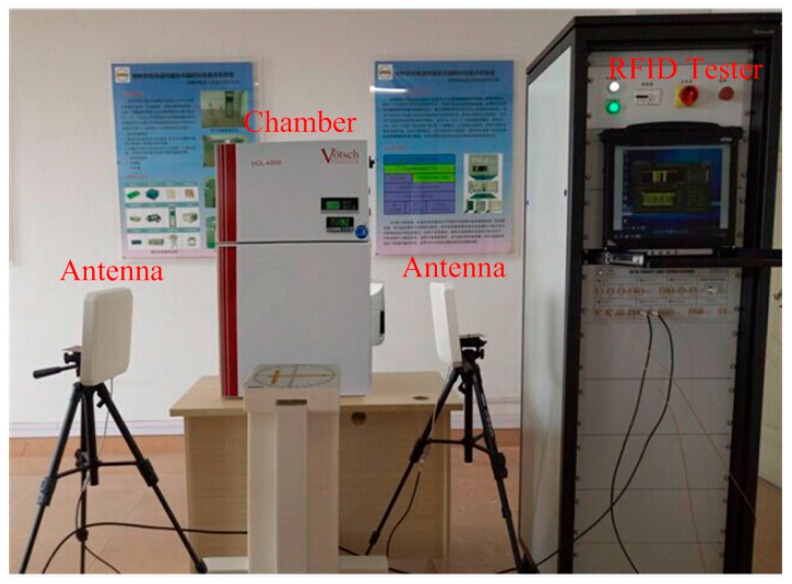
Wireless measurement environment.

Figure 14 shows the wireless testing environment for the proposed humidity sensor. The VISN-R1200 is a special RFID tester from VI Service Network, which can process, analyze, and display the testing signals simultaneously. The sensor performance was measured in a temperature and humidity chamber of Votsch VCL4003. The temperature range and resolution of VCL4003 are −40–180 °C and 0.1 °C respectively, the humidity range and resolution of VCL4003 are 10%–98% RH and 1% RH, respectively.

**Figure 15:**
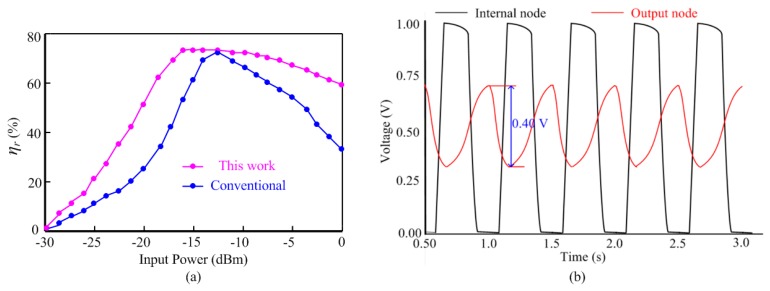
(**a**) Measured power conversion efficiency of the rectifier; (**b**) output waveforms of the proposed ring oscillator at different nodes.

The measured *η_r_* curve of the proposed rectifier is shown in Figure 15a. As compared to the conventional differential-drive rectifier [36], the proposed rectifier shows a flat *η_r_* curve. When input power is 13 dBm, the two curves both reach the optimal point 69%. As for the input power range whose *η_r_* is above 60%, the proposed rectifier and the conventional rectifier achieve −19 dBm and −7 dBm range, respectively. Figure 15b illustrates that the output node of the clock generator swings between the supply voltage 1.0 V and the ground, while the internal nodes of the clock generator vary from 0.3 V to 0.7 V. The reduction of the voltage swing to nearly 50% of its nominal value lowers the dynamic power consumption of the internal nodes by 75% and the overall power consumption by much more than 25%.

**Figure 16:**
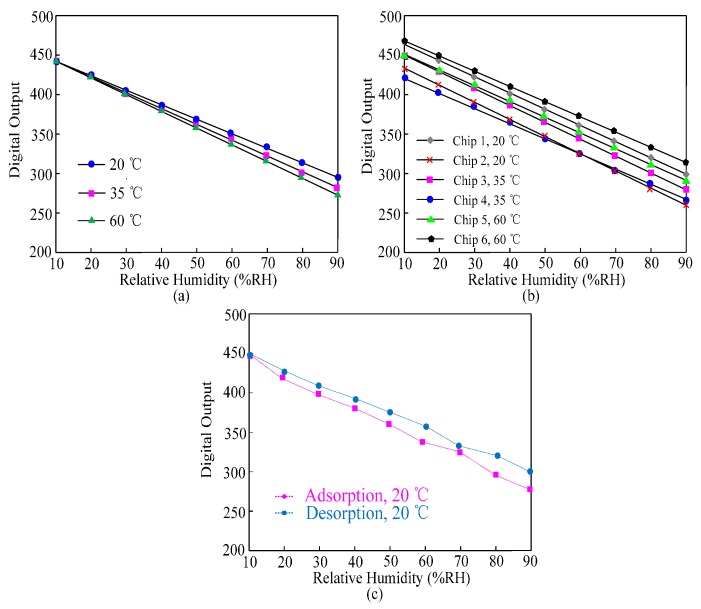
Measured humidity performances of the wireless sensor: (**a**) digital outputs at 20 °C, 35 °C, and 60 °C; (**b**) digital outputs of five test chips at 20 °C, 35 °C, and 60 °C; (**c**) hysteresis performances at 20 °C.

Figure 16a shows the twice measured results of the sensor tag at 20 °C, 35 °C, and 60 °C respectively within the relative humidity (RH) range from 10% to 90%. The sensor tag achieves high linearity and shows a maximum error of 12% for 40 °C offset. The temperature dependence of the dielectric constant of the polyimide film *ε_wet_* results in this error. Figure 16b shows the measured results of six test chips at 20 °C, 35 °C, and 60 °C within the range from 10% RH to 90% RH. Due to the process variation, the chips exhibit different digital outputs for a given RH value but all the results show an excellent linearity. The average sensitivity is 0.55% RH. The hysteresis performance of the sensor at 20 °C is shown in Figure 16c. The maximum difference between the moisture adsorption and desorption at the point 80% RH does not exceed 10%.

Table 2 compares the performances of the proposed RFID humidity sensor with previous RFID humidity sensor. The chipless RFID humidity sensor [18] is specially designed for ultra-low cost application, however it has the lowest accuracy because there is no digital block in it. The wireless humidity sensor with 13.56 MHz operating frequency [19] can obtain a high power conversion efficiency, however its operating distance is very limited. A RFID-based temperature and humidity sensor [20] can achieve complex function, however its operating distance is only 2.7 m. The proposed wireless humidity sensor achieves a minimum power dissipation of 5.7 μW, resulting in a maximum operating distance of 17 m in free space. Our design is especially for the tough sensing application in concrete.

**Figure 17:**
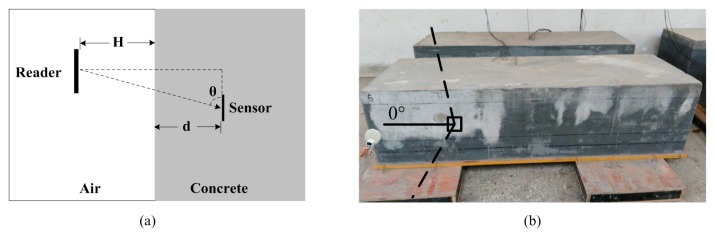
(**a**) Experimental scheme; (**b**) Test site.

Figure 17a shows the experimental scheme of the proposed wireless sensor in concrete. The depth of the wireless humidity embedded in concrete is *d*, the distance of the RFID reader placed above the concrete is *H*, the incidence angle between the reader and the sensor is *θ*. The photo of the test site is shown in Figure 17b.

**Figure 18:**
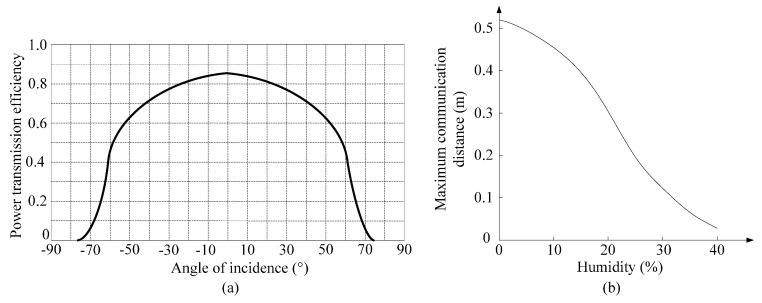
(**a**) Influence of incidence angle on power transmission; (**b**) Influence of concrete humidity on power transmission.

We embedded the sensor into the concrete at a depth of 8 cm and then investigated the influence of incidence angle on power transmission. The relationship between the incidence angle *θ* and power transmission is shown in Figure 18a. From that, we can see the minimum power loss is achieved when *θ* is 90°, the power transmission efficiency is 85%. When *θ* is larger than 75°, the power transmission efficiency is nearly 0%. Hence, in order to achieve the best test results, the incidence angle should be as vertical as possible. When we set *θ* = 90° and *d* = 8 cm, we measure the maximum communication distance of RFID reader. A calibrated humidity sensor SHT75 is embedded together with the proposed wireless humidity sensor to monitor the concrete humidity. For each measurement, the reader was instructed to perform 1000 attempts to read the sensor data from the wireless humidity sensor. When the success ratio remains above 80%, the communication distance is considered to be reliable. As shown in Figure 18b, the measured maximum communication distance decreases when the concrete humidity increases. The maximum communication distance is 0.52 m or 0.11 m respectively when the concrete humidity is 0% or 30%.

**Figure 19:**
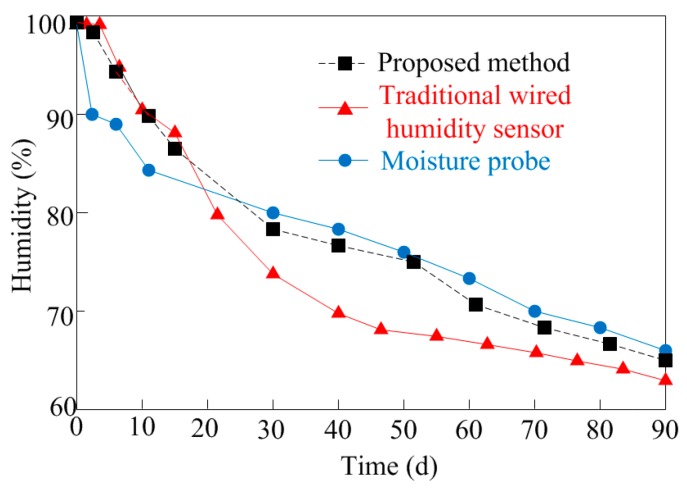
Performances comparison measured by different sensors.

This work employs concrete made of normal aggregate (1 h pre-wetting, moisture content of 6.6%) as the sample. The proposed wireless humidity sensor is embedded at a depth of 8 cm inside the concrete sample with the calibrated wired humidity sensor SHT75. The results measured by these two sensors compared with results of the moisture probe are plotted in Figure 19. According to [41], during the early age of concrete maturing, the traditional wired humidity sensor can get more accurate results in the first 14 days resulting from the exchange of air when measuring by moisture probe. Due to the long time embedded in wet concrete, zero drift of the traditional wired humidity sensor will decrease the accuracy of the measurement results. Thus, after the 14th day the moisture probe achieves higher measurement accuracy. From Figure 18 we can see in the first 14 days the proposed wireless sensor achieves nearly the same results with the traditional humidity sensor. After that, the measurement results of the proposed sensor are close to the results measured by the moisture probe. Hence the proposed wireless humidity sensor achieves better performances during the entire measurement.

## 5. Conclusions

Humidity monitoring plays an important role in concrete measurement, and thus this work presents a wireless humidity sensor based on UHF RFID technology. Considering the high losses of electromagnetic waves in concrete, a patch antenna is proposed to ensure the sensor tag can work inside of the concrete. The wireless humidity sensor employs a novel architecture and is carefully designed for low power application. The measured results show the proposed sensor tag achieves high humidity linearity with a normalized sensitivity of 0.55% %RH at 20 °C. The maximum operating distance is 0.52 m when the proposed wireless sensor is embedded into the concrete at a depth of 8 cm. The measured results are highly consistent with the results measured by traditional methods. The whole experiment demonstrates that the proposed wireless humidity sensor can provide reliable performance.

## Figures and Tables

**Table 1:** Design parameters of the patch antenna.

Wp	Lp	Ls1	Ls2	Ws1	Ws2	Ws3	Lf1	Lf2	S
42 mm	53 mm	23 mm	35 mm	32 mm	29.5 mm	1.7 mm	15 mm	3 mm	2.5 mm

**Table 2:** Performances comparison of various type RFID humidity sensor.

Design	Frequency	Normalized Sensitivity	Inaccuracy	Distance	Humidity Range	Cost
[18]	900 MHz	1.1%	9.2%	No	20%–70%	Ultra low
[19]	13.56 MHz	0.8%	4.7%	0.03 m	15%–85%	Low
[20]	900 MHz	0.7%	4.5%	2.7 m	20%–80%	Low
This work	915 MHz	0.55%	3.8%	17 m	10%–90%	Low
